# Development and Evaluation of Cefadroxil Drug Loaded Biopolymeric Films Based on Chitosan-Furfural Schiff Base

**DOI:** 10.3797/scipharm.0912-20

**Published:** 2010-09-26

**Authors:** Ritu B. Dixit, Rahul A. Uplana, Vishnu A. Patel, Bharat C. Dixit, Tarosh S. Patel

**Affiliations:** 1 Ashok & Rita Patel Institute of Integrated Study & Research in Biotechnology and Allied Sciences, New Vallabh Vidyanagar-388121, India; 2 Department of Pharmaceutics, A. R. College of Pharmacy, Vallabh Vidyanagar-388120, India; 3 Department of Chemistry, V. P. & R. P. T. P. Science College, Vallabh Vidyanagar-388120, India

**Keywords:** Cefadroxil, Biopolymeric films, Schiff base of chitosan-furfural, *In vitro* drug release

## Abstract

Cefadroxil drug loaded biopolymeric films of chitosan-furfural schiff base were prepared by reacting chitosan with furfural in presence of acetic acid and perchloric acid respectively for the external use. Prepared films were evaluated for their strength, swelling index, thickness, drug content, uniformity, tensile strength, percent elongation, FTIR spectral analysis and SEM. The results of *in vitro* diffusion studies revealed that the films exhibited enhanced drug diffusion as compared to the films prepared using untreated chitosan. The films also demonstrated good to moderate antibacterial activities against selective gram positive and gram negative bacteria.

## Introduction

Chitosan is a modified naturally occurring carbohydrate polymer derived from chitins which are found in crustaceans, fungi, insects and some algae [1–4]. Chitosan can be used in the diverse fields ranging from flocculent for seed coating, toiletry components, controlled drug delivery systems [5], membrane based transdermal drug delivery systems [6–14], wound-healing, dressing material and artificial skins etc. Chitosan having high molecular weight has been reported to have good film-forming properties [15] due to its intra- and intermolecular hydrogen bonding [6, 16–18]. The characteristics of chitosan film varied from one report to another might be due to difference in the source of chitin used to prepare chitosan, as well as type of solvents, methods of film preparation, and type and amount of plasticizers used, also affect the quality of the chitosan films [16]. There are reports about schiff base of chitosan and its film forming ability because of its potentiality in the field of prolonged drug delivery system and in some other type of novel drug delivery systems [19–22]. Moreover, cefadroxil is a broad spectrum antibiotic that acts against an extensive variety of bacteria, including Gram-positive and Gram-negative bacteria. Cefadroxil belongs to a group of cephalosporin antibiotics, which are used to treat many different types of bacterial infections [23]. However, Schiff base prepared by reacting chitosan and furfural has not been reported so far. Therefore, the present work aimed to develop and evaluate the cefadroxil loaded biopolymeric films of chitosan-furfural Schiff base by the reaction of amino group of chitosan with aldehyde group of furfural.

## Materials and Methods

### Materials

Chitosan with a molecular weight 4.5 × 10^5^ g/mol, degree of deacetylation 78% and viscosity 225 cp was obtained as a gift sample from Fish Processing Division, Central Institute of Fisheries Technology, Cochin, Kerala, India, and was used after purification. Furfural was purchased from LOBA chemicals, India. Glacial acetic acid was purchased from National Chemicals, India. Cefadroxil obtained as a gratis sample from Southern Pharmaceutical Limited, Baroda, India. All other chemicals used were of analytical reagent grade.

### Methods

#### Preparation of Cefadroxil Loaded Plain Chitosan Film (F_1_)

Chitosan film was prepared by following the reported method [24]. Accordingly, 1 g chitosan was dissolved in 12.8 mL acetic acid solution (1% v/v) by vigorous agitation for 30 minutes. The solution containing vessel was immersed in boiling water bath for 10 minutes, cooled down to room temperature and solution was filtered through glass-wool to remove undissolved particles of chitosan. The resultant solution was further stirred on magnetic stirrer at 200 rpm and at 37 °C for 8 hours. After that, 3.2 mL glycerol (20% v/v) as a plasticizer and 30 mg of cefadroxil were added in the solution, with continuous stirring until the drug completely dissolved in the solution. Subsequently, 15 mL film casting solution was casted onto a petri-dish (100 × 10 mm) precoated with polyurethane and was kept in an oven at 37 °C for complete drying. Prepared film was washed with 50% v/v ethanol to remove surface bound traces of glacial acetic acid. Dried film was carefully peeled out and stored in desiccator containing saturated sodium bromide.

#### Preparation of Cefadroxil Loaded Chitosan-Furfural Films (1:1 and 1: 0.5) (F_2_ & F_3_)

Chitosan-furfural film was prepared by following the reported method, using different mole ratio of chitosan to furfural [25]. Accordingly, 1 g chitosan was dissolved in 6.4 mL acetic acid solution (1% v/v) by vigorous agitation for 30 minutes. The solution containing vessel was immersed in boiling water bath for 10 minutes, cooled to room temperature and solution was filtered through glass-wool to remove undissolved particles of chitosan. The filtrate was treated with 6.4 mL furfural solution (1 mL furfural dissolved in 5 mL isopropyl alcohol). The mixture was stirred continuously for 8 hours at 37 °C. After that 3.2 mL glycerol (20% v/v) as a plasticizer and 30 mg of cefadroxil were added in the resultant solution by continuous stirring until formation of clear solution. Subsequently, 15 mL solution was casted onto a petri dish, precoated with polyurethane and kept it in oven at 37 °C for complete drying. Prepared film was washed with 50% v/v ethanol to remove surface bound traces of acid. Dried film was carefully peeled out and stored in desiccator containing saturated sodium bromide until further tests.

#### Preparation of Chitosan-Furfural (1:1) Film in Presence of Perchloric Acid (F_4_)

Cross-linked chitosan–furfural film was prepared by the method reported in the literature [26]. Accordingly, 1 g chitosan was dissolved in 6.4 mL acetic acid (1% v/v) solution by vigorous agitation for 30 minutes. The solution containing vessel was immersed in boiling water bath for 10 minutes, cooled down to room temperature, and solution was filtered through glass-wool to remove undissolved particles of chitosan. To this solution, 0.05 mL perchloric acid was added drop by drop at 0 °C temperature by keeping mixture containing vessel in ice bath. Then, 6.4 mL furfural was added (1 mL furfural was dissolved in 5 mL isopropyl alcohol) under continuous stirring for 45 minutes. After that 3.2 mL glycerol (20% v/v) as a plasticizer and 30 mg cefadroxil were added in the resultant solution by continuous stirring until the formation of clear solution. Subsequently, 15 mL solution was casted onto a petri-dish precoated with polyurethane and content was dried in a vacuum oven. Produced film was washed with 50% v/v ethanol to remove surface bound traces of acid. Dried film was carefully peeled out and stored in desiccator containing saturated sodium bromide until further tests. The compositional data for all the film formulations are presented in Table 1.

#### Physical and Mechanical Characterization of Films

The conventional and modified chitosan films were evaluated for their physical properties like thickness, percent elongation, tensile strength, folding endurance, water content, surface pH and SEM. The formation of schiff base during the reaction was confirmed by IR spectral analysis.

#### Thickness of the Films

A micrometer was used to measure the thickness of the film with least count of 0.001 mm prior to all the tests. Mean of five measurements across each film specimen was determined. To determine weight uniformity of the each film, five specimens of size 2.0 cm of all films were weighed on electronic balance and mean weight was calculated.

#### Tensile Strength and % Elongation

Tensile strength was evaluated using an Instron Universal Testing instrument (Model 4206, Instron Ltd., Japan) with a 2 kg load cell. Film of the required dimension without any air bubbles or physical imperfections was held between two clamps positioned at a distance of 3 cm. During the measurement, the top clamp was pulled at a rate of 100 mm/minutes and the force and elongation were measured upon breaking the films. The results from film sample that broke down between the clamps were used. Measurements were run in triplicate for each film. Tensile strength and percent elongation were calculated by applying the following equations:

$$\begin{array}{c} \text{Tensile}\;\text{strength}\;=\frac{\text{Force}\;\text{at}\;\text{break}\;(N)}{\text{Initial}\;\text{cross}\;\text{sec}\;\text{tional}\;\text{area}\;\text{of}\;\text{the}\;\text{sample}}\\ \%\;\text{Elongation}=\frac{\text{Increase}\;\text{in}\;\text{length}}{\text{Original}\;\text{length}}\times 100 \end{array}$$

#### Folding Endurance Test

The folding endurance of the film was determined by repeatedly folding one film at the same place till it broke down or folded up to, which is considered satisfactory to reveal good film properties [27]. The number of times the film could be folded at the same place without breaking gave the value of the folding endurance.

#### Equilibrium Water Content and Swelling Ratio of the Films

All the prepared films were tested by placing each film in 30 mL distilled water and incubated at 37 °C. At an appropriate time interval, the films were taken out and excess water was removed from the film carefully using filter paper and films were weighed immediately. The swelling of film was expressed as a swelling ratio (SR) using following equation:

$$SR=\frac{{\text{W}}_{\text{t}}-{\text{W}}_{0}}{{\text{W}}_{0}}$$

Where : W_0_ is the weight of dry sample (g) and W_t_ is the weight of the sample (g) at time ‘t’ after incubation for 30 minutes.

#### Surface pH

To measure the surface pH, prepared films were left to swell for 2 hrs. in double distilled water with stirring. The surface pH was measured by means of a pH paper (strip) placed on the surface of the swollen film. The mean of two readings was duly recorded.

#### Infrared Spectroscopy

Infrared spectra of all the films were carried out using KBr disc technique on Perkin-Elmer Spectrum GX FT-IR model at SICART, Vallabh Vidyanagar.

#### Differential Scanning Calorimetry

DSC curves for all the prepared films were obtained using the Mettler TA 3000 DSC apparatus. The film was heated from 50 °C to 450 °C at a heating rate of 10 °C/minutes, at SICART, Vallabh Vidyanagar.

#### Scanning Electron Microscope (SEM) Study

The morphology and surface topography of the film was examined by SEM using model JSM-840 A, SEM–Jeol, Japan, at E.R.D.A., Makarpura, GIDC, Baroda. Spherical samples (5 mm to 2 mm) were mounted on the SEM sample stab using a double-sided sticking tape. The samples were coated with platinum (200 A°) under reduced pressure (0.001 torr) for 2 minutes using an ion sputtering device (model JFC-1100 E, Jeol, Japan). The platinum-coated samples were observed under the SEM and photomicrographs of suitable magnifications were obtained.

#### Determination of Drug Content

The concentration of the cefadroxil in all the films was determined by spectrophotometry [28]. Film was weighed accurately and quantitatively transferred into a 100 mL volumetric flask, which was diluted up to the mark with distilled water. This mixture was stirred for 24 hrs. to allow the total release of the drug from the film. After filtration the filtrate was assayed using ultraviolet spectrophotometer at the wavelength of maximum absorbance. The assay was performed in triplicate. The percentage of the drug content of the film was calculated by the following equation:

$$\text{Drug}\;\text{Content}\;(\%w/w)=\frac{\text{mass}\;\text{of}\;\text{drug}}{\text{mass}\;\text{of}\;\text{film}}\times 100$$

#### In Vitro Drug Release Study Dissolution Studies

Each type of film was introduced into each of three dissolution vessels (i.e. in triplicate) and the dissolution was determined according to the USP (1995:2330) apparatus type II (paddle). After withdrawal of a 5 ml sample, it was immediately replaced with 5 ml of preheated dissolution medium. The samples were filtered through a 0.20 *μm* membrane and then analyzed with a UV spectrophotometer at the wavelength of maximum absorbance (263 nm). The operating conditions for the dissolution tests are summarized in Table 2.

#### Antibacterial Activity

The bacteriostatic effect of the films containing cefadroxil was determined by agar-plate diffusion assay [29]. A cotton swab charged with *Staphylococcus aureus* (ATCC 25923), *klebsiella pneumoniae* (ATCC 13883)*, Escherichia coli* (ATCC 25922) and *Proteus vulgaris* (ATCC 13315) suspension (106 CFU/mL) were inoculated on plates and bacteria was spread evenly over the surface of the Luria agar media. Then, films (1 cm × 1 cm) were placed on inoculated agar-plates. These plates were incubated at 37 °C for 24 hrs. and the zone of inhibition were measured and recorded. The films were removed individually from the plates, dabbed on a sterile swab to remove surface moisture, and immediately transferred to another fresh plate inoculated with the same bacteria. This procedure was repeated until no zone of inhibition was seen (> 10 mm).

## Results and Discussion

### Physical and Mechanical Characterization of Films

Chitosan containing free amino group was reacted with furfural under two different reaction conditions. In the presence of acetic acid as a catalyst, amino group of chitosan was reacted with aldehyde group of furfural to produce light yellow coloured biopolymeric Schiff base [19] films (F_2_ and F_3_). While, in the presence of perchloric acid as a catalyst, furfural ring might have been opened up to form aliphatic Schiff base [30, 31] with two different chitosan repeating units to produce cross-linked dark yellow coloured biopolymeric film (F_4_). Composition of various cefadroxil drug loaded chitosan films are shown in Table 1 and their physical and mechanical properties are included in Table 3. The mean thickness of the film formulations was measured and found within the range of 0.08 ± 0.02 to 0.13 ± 0.02 mm. The weight of different film formulation was measured and found within the range of 0.99 ± 0.05 gm to 1.25 ± 0.09 gm. There was no significant variation in the weight of the films. The result shows that the method of preparation of films was consistent. Folding endurance test results indicated that none of the film breaks until it is folded 170 times, which showed that the films would not broke down and that they maintain their integrity with general skin folding when applied. There was an influence of the film composition on the swelling of the chitosan films. However, an increase in weight was not observed even after keeping films in water up to 24 hrs. Whereas, changes in the diameter of the F_1_ film to greater expansion and swelling was observed, this behavior is associated with the solubility of chitosan-acetate in water. On the other hand F_2_ and F_3_ films formulations showed lower swelling ratio in comparison of F_4_ film due to cross-linking of chitosan with furfural. Tensile strength of all the film formulations was within the range of 1.12 to 1.44 kg/mm^2^ and % elongation was within the range of 29.0% to 33.0%. The results of tensile strength and % elongation were found to be satisfactory for all the formulations. The F_4_ film formulation showed highest tensile strength and the F_1_ film formulation showed lowest tensile strength. On the other hand elongation was greatest for F_1_ film compared to all other films.

### Infrared Spectroscopy

**Fig. 1.**
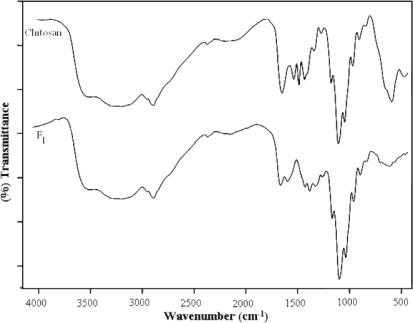
FT-IR spectra of Chitosan and F_1_ film

**Fig. 2.**
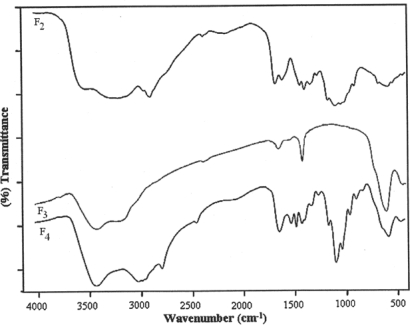
FT-IR spectra of F_2_ to F_4_ films

Investigation of IR spectral data of chitosan films showed a broad band around 3490 cm^−1^ due to presence of –OH and –NH_2_ groups. Most significant part of the spectrum was bands appeared at 1668 and 1320 cm^−1^ due to amide group. There were characteristic bands at 1462, 1415, 1378, 1178 and 810 cm^−1^, in addition to the usual broad band at 2880 cm^−1^, which are in good agreement with the reported IR spectrum of chitosan [32–34]. The reaction of chitosan with furfural forms the biopolymeric Schiff base. The IR spectra of Schiff base biopolymeric films showed a strong absorption band at 3360 cm^−1^ for the –OH group. The band at 1410 and 1633 cm^−1^ may be attributed to the C=N vibration characteristics of imine group [13, 14]. On the other hand, there was no evidence for the presence of aromatic aldehyde group of furfural near 1670 cm^−1^ in the IR spectrum of Schiff base polymer. The IR spectrum confirms the imine formation represented by the absorption band in the region of 1540–1590 cm^−1^, which was not observed in the original chitosan spectrum. Moreover, the band appeared at 1096 and 1251 to 1320 cm^−1^ in the spectrum of cross-linked film shows the presence of 2° alcohol [30, 31]. It might be due to opening of furfural ring during formation of Schiff base, which confirms the cross-linking of furfural with the two units of chitosan. The IR spectra of all four films are shown in Figures 1 and 2.

### Differential Scanning Calorimetry

**Fig. 3.**
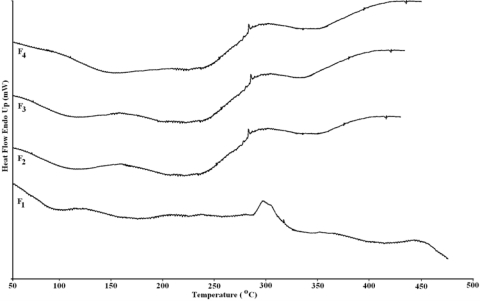
Differential Scanning Calorimetry Spectra of F_1_ to F_4_ films

DSC data of all the films are included in Table 4 and DSC spectra are shown in Figure 3. Scrutinization of DSC spectra shows that F_1_ film of chitosan exhibits two endothermic peaks at 90 and 289 °C and two exothermic peaks at 310 and 456 °C, which are very close to the reported data of chitosan [35]. Similarly, DSC data of F_2_, F_3_ and F_4_ films were also in close agreement with the reported data of chitosan, but shows absence of fourth stage exothermic peak. This confirms the structural modification of chitosan upon addition of furfural, which may result in to a linear or cross-linked biopolymeric films depending upon the reaction condition.

### Scanning Electron Microscope Study

**Fig. 4.**
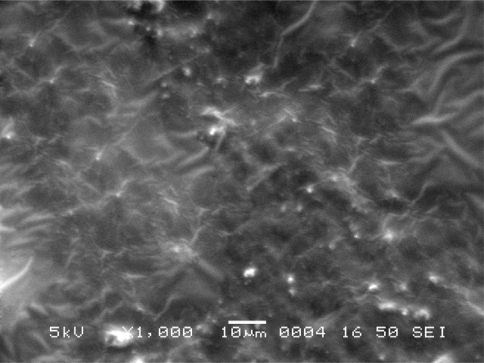
Scanning electron microscope photograph of F_1_ film formulation.

**Fig. 5.**
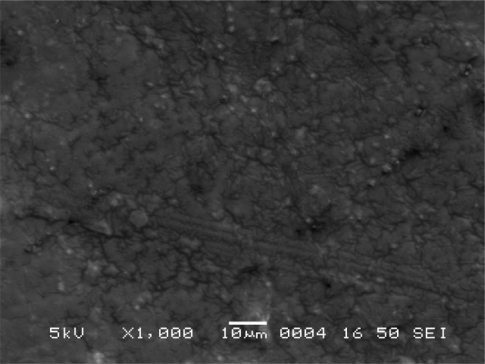
Scanning electron microscope photograph of F_2_ film formulation.

**Fig. 6.**
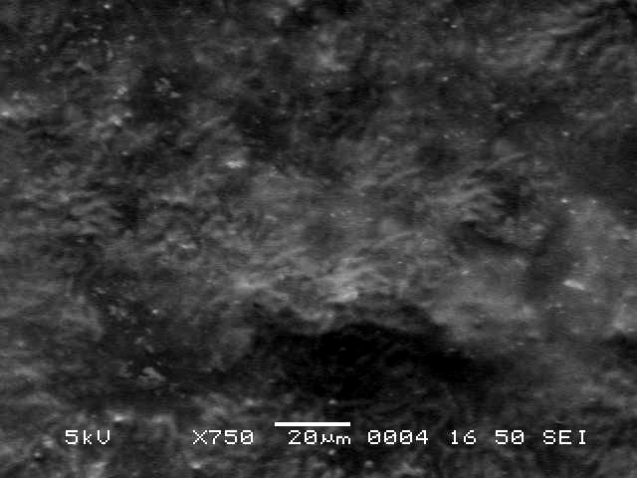
Scanning electron microscope photograph of F_3_ film formulation.

**Fig. 7.**
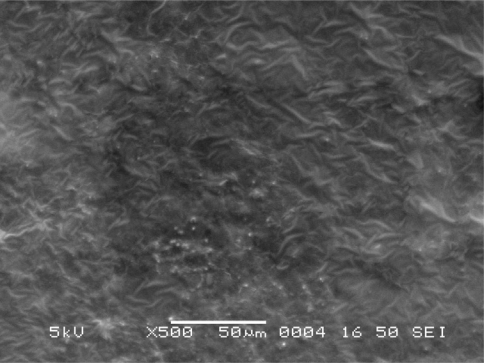
Scanning electron microscope photograph of F_4_ film formulation.

Scanning electron microscopic (SEM) study of different film formulations was performed and the SEM results of films F_1_ to F_4_ are shown in Figures 4 to 7 respectively. Observation of SEM of film F_1_ showed voids on the surface, while in case of F_2_, F_3_ and F_4_ films such kind of voids were not observed. Absence of voids in the F_2_, F_2_ and F_4_ films might be due to the cross-linking of chitosan with furfural.

### Drug Content

The drug content uniformity among the different films was observed in the range of 27.00 to 29.00 mg/film. The results indicate that the process employed to prepare films in the present work is capable of producing films with uniform drug content with minimum variation. Thus, the results showed that all the films having drug content uniformity within the acceptable range.

### In vitro Permeation Studies using Dialysis Membrane

The diffusion cell was fabricated with the help of small funnel. The donor compartment was funnel of dish size 3.5 cm diameter. The film formulations F_1_, F_2_, F_3_ and F_4_ of 1.5 cm^2^ surface areas were placed on dialysis membrane individually with aluminum foil as membrane, which was then tied to the diffusion cell. This diffusion cell was immersed in beaker containing 50 ml of phosphate buffer (pH 7.4) [36] and methanol (50% v/v) as diffusion media receptor compartment. The solution was stirred using magnetic stirrer at the speed of 100 rpm by maintaining the temperature of the whole assembly at 37 °C. The amount of the drug release was determined by withdrawing 2 ml of sample at specific time interval up to 12 hrs. The volume withdrawn was replaced with equal volume of fresh and pre-warmed phosphate buffer media containing methanol. The absorbance of the withdrawn sample was measured at 240 nm to estimate cefadroxil [37].

### In Vitro Drug Release Study

**Fig. 8.**
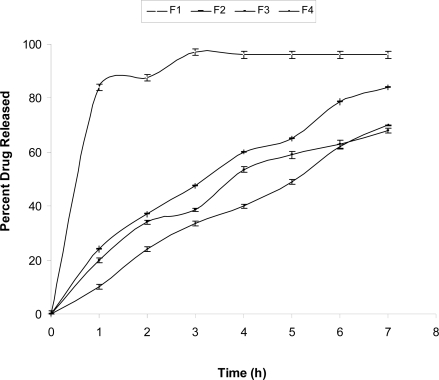
*In vitro* drug release profile of percent drug release Vs time for different film formulations. [Graph shows S.D. of triplicate experiments]

**Fig. 9.**
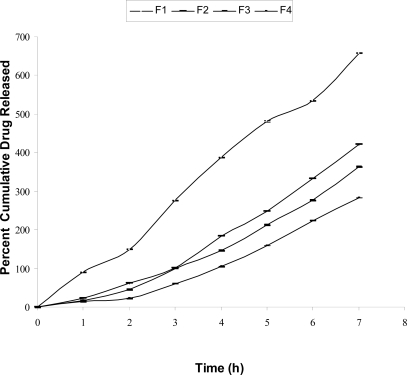
*In vitro* drug release profile of percent cumulative drug release Vs time for different film formulations. [Graph shows S.D. of triplicate experiments]

The film formulations F_1_, F_2_, F_3_ and F_4_ loaded with cefadroxil were subjected to *in vitro* drug release study using dissolution test apparatus type-II. The results of percent drug release of films at different time point is shown in Figure 8, while percent cumulative drug release is shown in a Figure 9. Scrutinization of Figures 8 and 9 showed that in F_1_ film, a rapid drug release was observed, while in F_2_ and F_3_ films drug released at moderate rate. This might be due to the formation of schiff base between chitosan and furfural. F_4_ film showed slow drug release which might be due to entrapment of cefadroxil drug molecules between cross-linked chitosan polymeric chains.

The drug release data for different batches was analyzed using PCP DISSO software [38] version 3.0, developed by Poona College of Pharmacy, Pune, India. Table 5 lists various dissolution kinetics parameters computed for all the four films (F_1_ to F_4_). In the present study, all four formulations followed zero order release pattern (Figure 8).

### Antibacterial Activity

The antibacterial activity of different film formulations were carried out against *S. aureus, K. pneumoniae* (Gram positive) and *E. coli*, *P. vulgaris* (Gram negative) bacteria of different films. To measure the zone of inhibition of different film formulations F_1_, F_2_, F_3_ and F_4_, experiments were performed in triplicates and the average value of results is summarized in Table 6. Results of antibacterial activity showed that *S. aureus, K. pneumoniae* and *E. coli* have good to moderate activity as compare to *P. vulgaris* bacteria. Moreover, prolonged drug release was observed for the films F_2_, F_3_ and F_4_ as compare to F_1_ film.

## Conclusion

Novel biopolymeric schiff base films were developed for the release study of cefadroxil drug, which can be used for external application in the form of dermal patches. Modified chitosan films have better physical properties and mechanical strength as compare to chitosan films. Drug release study of the plain chitosan film and modified chitosan films were compared and it was observed that modified chitosan films shows better release profile than plain chitosan films. Drug loaded modified chitosan films showed significant antibacterial activity as compare to chitosan drug loaded film.

## Figures and Tables

**Tab. 1.** Composition of Various Cefadroxil Drug Loaded Chitosan Films^a^

**Formulation Code**	**Chitosan (% w/v)**	**Furfural (%v/v)**	**Glycerol (%)**	**Drug (mg)**
F_1_ – Chitosan in acetic acid	1.0	–	20	30
F_2_ – Chitosan-furfural (1:1) film in presence of acetic acid	1.0	1.0	20	30
F_3_ – Chitosan-furfural (1:0.5) film in presence of acetic acid	1.0	0.5	20	30
F_4_ – Chitosan-furfural (1:1) film in presence of perchloric acid	1.0	1.0	20	30

a 15 mL film casting solution was used for the preparation of film.

**Tab. 2.** Operating Parameters for dissolution test apparatus type II

**Operating parameter**	**Value**
Rotation speed	100 rpm
Dissolution medium	Water
pH of dissolution medium	7 pH
Volume of dissolution medium	900 ml
Temperature	37 ± 0.5 °C
Pre-determined time intervals for sampling	60, 120, 180, 240, 300, 360, 420 min.
Sample volume	5 ml
Wavelength	263 nm
Filter membranes	0.2 μm

**Tab. 3.** Evaluation of Chitosan Films

**Sr. no.**	**Parameters**	**Film Formulation Code**			
		**F**_**1**_	**F**_**2**_	**F**_**3**_	**F**_**4**_
1	Thickness (cm)	0.08 ± 0.02	0.11 ± 0.02	0.12 ± 0.02	0.13 ± 0.02
2	Mass of 2.0 cm Diameter Films (gm)	0.99 ± 0.05	1.08 ± 0.01	1.23 ± 0.02	1.25 ± 0.09
3	Tensile Strength (kg/mm^2^)	1.12 ± 0.01	1.21 ± 0.01	1.19 ± 0.01	1.44 ± 0.01
4	(%) Elongation	32.89 ± 0.85	31.02 ± 1.77	29.04 ± 1.74	28.96 ± 1.66
5	Surface pH	5.70	5.90	5.93	5.97
6	Swelling ratio of films (gm/gm)	1.11 ± 0.08	0.86 ± 0.10	0.75 ± 0.10	0.91 ± 0.07
7	Cefadroxil Content (mg/film)	28.19 ± 0.22	27.87 ± 0.62	27.82 ± 0.57	27.91 ± 0.51

(Presented values: mean ± S.D., n=3)

**Tab. 4.** DSC Thermograms of Biopolymeric Films

**Film**	**First Stage**	**Second Stage**	**Third Stage**	**Fourth Stage**

	**Peak Temp. (°C) (Endothermic heat flow)**		**Peak Temp. (°C) (Exothermic heat flow)**	
F_1_	90	289	310	456
F_2_	98	280	400	–
F_3_	98	282	412	–
F_4_	102	285	439	–

**Tab. 5.** Dissolution Kinetics Parameters for the films F_1_ to F_4_

**Film Formulation**	**N**	**K**	**r^2^**
F_1_	0.9844	14.24	0.9839
F_2_	0.7954	15.55	0.9858
F_3_	0.9494	55.40	0.9895
F_4_	0.8900	30.50	0.9019

**Tab. 6.** Zone of Inhibition Value (cm) of Different Film Formulations against Various Bacterial Species

**Days**	S. aureus*						K. pneumoniae*					

	**F**_**1**_	**F**_**2**_	**F**_**3**_	**F**_**4**_	**F**	**G**	**F**_**1**_	**F**_**2**_	**F**_**3**_	**F**_**4**_	**F**	**G**
1	1.0	0.3	0.5	0.7	0.4	0.3	1.1	0.6	0.8	0.9	0.4	0.5
2	1.1	0.9	1.1	1.3	0.7	0.6	1.5	0.9	1.1	1.2	0.6	0.6
3	1.5	1.7	1.9	1.7	1.0	0.9	1.7	1.1	1.4	1.5	0.9	0.8
4	1.5	1.9	2.1	2.0	1.1	1.1	1.9	1.3	1.7	1.7	1.2	1.3
5	1.5	2.2	2.3	2.2	1.2	1.2	1.9	1.9	1.9	2.0	1.3	1.3
6	1.5	2.7	2.3	2.2	1.4	1.3	1.9	2.1	2.3	2.1	1.3	1.3

**Days**	E. coli*						P. vulgaris*					

	**F**_**1**_	**F**_**2**_	**F**_**3**_	**F**_**4**_	**F**	**G**	**F**_**1**_	**F**_**2**_	**F**_**3**_	**F**_**4**_	**F**	**G**

1	1.1	0.6	0.8	0.9	0.7	0.6	0.8	0.5	0.6	0.9	0.2	0.3
2	1.5	0.9	1.1	1.2	0.9	0.8	0.9	0.9	0.7	1.0	0.3	0.3
3	1.7	1.1	1.4	1.5	1.0	1.0	1.0	0.9	0.9	1.3	0.4	0.4
4	1.9	1.3	1.7	1.7	1.2	1.1	1.0	1.1	0.9	1.4	0.5	0.4
5	1.9	1.9	1.9	2.0	1.3	1.4	1.1	1.1	1.9	1.4	0.6	0.5
6	1.9	2.1	2.3	2.1	1.4	1.5	1.1	1.1	1.4	1.5	0.6	0.5

* Cefadroxil as standard and its zone of inhibition was 2.3 to 2.8 cm in 3 to 4 d; F…Furfural; G…Glycerol
